# In defense of sugar: a critical analysis of rhetorical strategies used in The Sugar Association’s award-winning 1976 public relations campaign

**DOI:** 10.1186/s12889-019-7401-1

**Published:** 2019-08-22

**Authors:** Cristin E. Kearns, Stanton A. Glantz, Dorie E. Apollonio

**Affiliations:** 1 Department of Preventive and Restorative Dental Sciences and Philip R. Lee Institute for Health Policy Studies, University of California, San Francisco, San Francisco, CA USA; 2 Department of Medicine, University of California, San Francisco, San Francisco, CA USA; 3 Department of Clinical Pharmacy, University of California, San Francisco, San Francisco, CA USA

**Keywords:** Sugar industry, Crisis communication, Corporate apologia, Non-communicable diseases

## Abstract

**Background:**

In 1976, the U.S. Sugar Association (SA), a globally networked trade organization representing the cane and beet sugar industry, won the Public Relations Society of America’s (PRSA) Silver Anvil Award for a crisis communication campaign. Their campaign successfully limited the diffusion of sugar restriction policies to control obesity, heart disease, diabetes, and dental caries, and marked the beginning of the modern-day SA. The sugar industry continues to resist measures to reduce sugar consumption, therefore understanding and addressing industry opposition is crucial to achieving global targets to reduce non-communicable disease.

**Methods:**

We critically analyze common crisis management rhetorical strategies used by SA to defend itself from perceived wrongdoing, and sugar from perceptions of harm using a thematic content analysis based on Hearit’s Corporate Apologia theory. Data sources were internal SA documents related to the 1976 Silver Anvil Award in 1) PRSA records, 2) Great Western Sugar Company records, and 3) William Jefferson Darby Papers.

**Results:**

SA, using prototypical apologia stances (counterattack, differentiation, apology, and corrective action) and rhetorical dissociation strategies (appearance/reality, opinion/knowledge, and act/essence) constructed a persuasive narrative to successfully defend sugar from a product safety crisis, and the sugar industry from a social legitimacy crisis. SA’s overarching narrative was that restricting sugar, which it claimed was a valuable food that makes healthy foods more palatable, would cause harm and that claims to the contrary were made by opportunists, pseudoscientists, food-faddists, lay nutritionists or those who had been misled by them. SA’s apologia does not meet criteria for truthfulness or sincerity.

**Conclusion:**

Corporate apologia theory provides an accessible way of understanding sugar industry crisis communication strategies. It enables public health actors to recognize and predict industry corporate apologia in response to ongoing product safety and social legitimacy challenges. Industry counterarguments can be examined for truthfulness and sincerity (or the lack thereof), and explained to policymakers considering sugar restriction policies, and to the public, thereby decreasing the effectiveness of illegitimate industry communication efforts to oppose regulation and legislation.

## Background

The World Health Organization’s (WHO) 2015 sugars guideline recommends that adults and children consume less than 10% of daily calories from free sugars, with a further reduction to 5% being ideal [1]. The guideline is part of the WHO’s efforts to reach targets set by the Global Action Plan for NCDs 2013–2020 to halt the rise in dental caries, diabetes, and obesity and reduce the burden of premature deaths due to NCDs by 25% by 2025 [2, 3]. In support of the WHO sugar guideline, effective, feasible policy actions exist for governments to reduce the availability and affordability of sugar and sugary products and raise awareness about the sugar contained in products [4]. However, food and beverage industry opposition to policies such as sugar-sweetened beverage taxes is a significant barrier that the public health community will need to overcome [5].

Investigators have begun utilizing a corporate political activity framework to analyze food and beverage industry influence on health, [6, 7] an approach modeled after systematic analyses of tobacco industry behavior [8, 9]. This framework is segmented into six strategies: information and messaging, financial incentives, constituency building, legal, policy substitution, and opposition and can be applied to understand and monitor food and beverage industry influence on health and reduce imbalances between public and commercial interests. Our current research expands on this initial work by examining sugar industry public relations activities through the lens of crisis communication literature.

Faced with extensive media coverage of negative health effects of sucrose consumption and regulatory threats from the U.S. Food and Drug Administration’s (FDA) pending review of the safety of sucrose in the 1970s, the U.S. Sugar Association (SA), a trade association representing the cane and beet sugar industry on sugar and health issues, together with the public relations firm Carl Byoir & Associates, Inc., won the Public Relations Society of America (PRSA) Silver Anvil Award in the category “Public Affairs – government relations/crisis management/media relations” in 1976 [10]. This campaign coincides with what SA describes on its 2018 website as “the start of the modern-day Sugar Association” [11]. Journalistic accounts of the SA Silver Anvil campaign have provided details about the strategies and tactics the industry used to successfully influence the FDA’s decision not to regulate sucrose as a food additive, which would have given the agency the power to limit the amount of sucrose added to processed foods, [12, 13] however there has been no formal analysis of the industry’s public relations response.

Crisis communications is both an organizational practice and an academic discipline [14]. Organizations experiencing a crisis, such as the sugar industry faced with charges that its product is unsafe, have an array of options when communicating with stakeholders, ranging from denying their product is guilty to accepting full responsibility [15]. This study used Hearit’s corporate apologia theory [16] as a framework to analyze the crisis communication strategies of SA. Hearit’s theory is inspired by the study of apologia, originally conceived as a defensive form of speech performed by individuals [17]. Drawing from organizational rhetoric, [18] social legitimacy theory, [19] and terminology typically associated with the remediation of guilt, [20] a corporate apologia is a distinct form of discourse applied in the midst of social legitimacy crisis to provide a persuasive counter narrative to perceived wrongdoing [21]. We chose Hearit’s theory because it provides a systematic way of understanding how the sugar industry develops arguments to respond to public criticism of its product and practices and normative guidelines to evaluate the ethics of these arguments. [16] Based on an ethical decision-making procedure known as casuistry, [16, 22] Hearit defines a clear-cut paradigm case of an ethical apologia to serve as a standard against which other cases can be measured. [14]

## Methods

This study relies on a content analysis of internal industry documents. Our initial data set included all documents SA submitted to PRSA for the 1976 Silver Anvil application, contained in the PRSA Records, 1938–2013, archived at the Wisconsin Historical Society (79 documents) [23]. We triangulated [24] these data with trade journals and additional documents related to SA’s public relations program in the 1970s located in the Records of the Great Western Sugar Company, Colorado Agricultural Archive, Colorado State University (47 documents), [25] and the William Jefferson Darby Papers, Eskind Biomedical Library Special Collections, Vanderbilt University Medical Center (27 documents) [26]. We used The *New York Times* to contextualize media criticism of sugar because it is the US “newspaper of record” [27].

Documents were entered into the QSR NVivo 11, and content coded based on Hearit’s corporate apologia theory (Table 1), including characteristics associated with an ethical corporate apologia paradigm case. CK did initial coding, then CK and DA jointly identified emerging impressions [30]. CK and DA then reviewed coded data extracts for each theme to ensure that themes accurately reflected the data [31].

**Table 1:** Crisis Response Analytical Framework Based on Hearit’s Corporate Apologia Theory [16, 21, 28, 29]

Crisis Type	Definition
Accidents	• Due to an act of God or a failure of human interaction with technology • Causes harm to innocents or the environment • Accident victims quickly secure legal counsel to pursue compensation for harms
Product Safety Incidents	• Product safety incidents tend to coalesce slowly as similar revelations from disparate sources begin to surface • Cause rooted in design flaws • Victims likely represented by legal counsel
Scandals and Illegalities	• Controversial or illegal activities that are likely to bring social sanction
Social irresponsibility	• An organization is accused of having committed acts that are incongruent with current social values
Crisis Response Type (Stance)	Definition
Denial	• An organization maintains that it has done nothing wrong. • A variant of this strategy is used by organizations that cannot deny committing but can deny intent.
Counterattack	• An offshoot of denial, an organization seeks to deal with the problem of its guilt by first denying the act and then directly attacking its accuser, claiming that the charges are false or come from malicious intent • The use of such a strategy reverses the direction of the exchange is reversed in that the accused takes the moral high ground position of the accuser, and seeks to put the interrogator on the defensive
Differentiation	• Organizations seek to distance themselves from their wrongdoing by attempting to redefine it, explain it, account for it, or justify it • The idea in doing so is that once key publics understand what happened, they will be less likely to condemn the organization and its actions. Such a strategy usually acknowledges some guilt, but often seeks to explain it away
Transference	• Organizations seek to achieve absolution by transferring it to another – scapegoating • The company is able to locate guilt, not in the company as a company, but instead in the actions of a few individuals, thus repairing the company’s social persona
Apology / Corrective Action	• Organizations deal with their misconduct by acknowledging it and confessing responsibility • Often coupled with a strategy of corrective action • Although some companies honestly and forthrightly issue a direct apology for their wrongdoing, most tend to release a statement of regret whereby they apologize for the harm that resulted but are careful not to assume responsibility
Legal	• An organizations accused of wrongdoing says nothing publicly, but instead takes a private, legal strategy, whereby they seek legal absolution of their guilt and ignore public concerns
Dissociation Types	Definition
Appearance / Reality	• An institution argues that there “appears” to be a perception that it is guilty of the alleged transgression • Asserts that the “true facts” reveal that in reality the organization is a law-abiding company that is not guilty of the alleged wrongdoing
Knowledge / Opinion	• Challenges the validity of the charges by redefining them as groundless • Asserts that critics’ claims are mere opinions and do not represent fact, thus bifurcating the previously unitary charge
Group / Individual	• A division is made by which guilt is transferred from the many to the one
Act / Essence	• Argue that although the act was committed, it was in no way representative of the essential quality of the company’s nature
Ethical Forms of Communication	Definition
Truthfulness	• An apologia should be characterized by a disclosure of useful information and not omit key facts that, when revealed, would fundamentally change how others view the apologist’s actions • An individual or an organization caught in a crisis should not engage in deception. If an apologist chooses to lie, such a choice should only be made as a last resort and then only for reasons that would survive public scrutiny—of which there are very few
Sincerity	• An individual or an organization must demonstrate a good-faith effort to achieve reconciliation • Must be rooted not just in operational performance but also in communicative performance • Must show evidence of a true desire to reconcile with offended stakeholders, rather than acting in such a way that it is evident that the apologist’s only desire is to escape from the media glare
Timely	• Performed as soon as the offender recognizes the offense
Voluntary	• It must be performed without actual or anticipated coercion • Communicates a sincere desire to reconcile, rather than an opportunistic attempt at damage control
Addresses all stakeholders	• Must speak to the concerns and interests of all parties who have been offended
Appropriate context	• Concerns the appropriateness of the site, location, or medium chosen

## Results

### Sugar and health crises facing the U.S. Sugar Association in the 1970s

SA’s 2-page Silver Anvil case summary for PRSA is in Table 2. The campaign addressed two concurrent, but distinct crises.

**Table 2:** Campaign Summary of The Sugar Association’s 1976 Public Relations Society of America Silver Anvil Award. [10, 32]

I. Problem: Sugar, because of its universal usage and visibility, was a natural, target for the lay nutritionists and promoters of fad foods and diets who appeared to capitalize on the concern generated by the consumer movement. Unfortunately, many well-meaning consumer advocates and lay writers were mislead [sic] by this promotional onslaught. In addition, the psychology of sweetness works against sugar, with critics maintaining it is a conditioned response. As a result, the industry faced a barrage of criticism in the media suggesting that public consumption of ever-increasing amounts of sugar was responsible for a far-ranging variety of health problems. The volume was sufficient to cause concern among the industry’s primary publics: the medical community, nutritional professionals, user industries, government health officials and the consuming public.	
II. Research: An extended survey of public attitudes by National Analysts, Inc. suggested that support for sugar was stronger than anticipated and that sugar’s primary publics would be receptive to the scientific facts. An independent survey of existing scientific data and literature search headed by Dr. F. J. Stare, chairman of Harvard’s Dept. of Nutrition, revealed sugar to be a safe food that in certain forms does contribute to dental caries. As well, it indicated that, the desire for sweet is inherent and that U.S. per-capita consumption had not changed in 50 years.	
III. Objectives/Short-Term: Reach the following target audiences with the scientific facts concerning sugar and enlist their aid in educating the consuming public: A. The medical community; B. Nutritional professionals; C. Sugar-using industries; D. The media; E. Government health officials. Objectives/Long-Term: To establish with the broadest possible audience- virtually everyone is a consumer—the safety of sugar as a food and that in moderation it plays an important role in a balanced diet.	
IV. Strategy: A. Drop all Assn. advertising; B. Move to a program of public information and education; C. Enlist the counsel of leading medical experts; D. Organize the scientific facts concerning sugar into cohesive documents; E. Let qualified medical experts speak for the industry, regardless of potential negatives; F. Respond immediately to all public criticisms; G. Seek media objectivity and balance, not necessarily objective coverage; H. Promote an understanding of sound basic nutrition; I. Stand up publicly against the purveyors of nutrition misinformation; J. Acknowledge sugar’s vulnerability in the area of dental caries and direct research to this problem.	
EXECUTION	
1. Fostered the organization of an independent Food & Nutrition Advisory Council (six doctors/two dentists) to counsel the industry and help it select appropriate medical research projects.	
2. Stimulated the research arm of the industry to fund recommended research projects, to learn more about sugar and provide additional information.	
3. Requested the FNAC to organize existing scientific facts concerning sugar in a series of papers. These were published” in *World Review of Nutrition and Dietetics* as “Sugar in the Diet of Man.” Distributed 25,000 reprints.	
4. Produced a 26-min film (“Nutrition’ Is”) on basic nutrition, for general distribution, that does not discuss sugar.	
5. Commenced a regional Nutrition Information Program, using free-lance dietitians, to take the sugar story to nutritional professionals.	
6. Appeared twice before the Newspapers/ Food Editors with a panel of doctors, once discussing sugar and health; once the crisis in nutrition information.	
7. Distribute literature to food editors and science writers.	
8. Respond to all public criticisms of sugar; send literature.	
9. Support university and professional information campaigns against individuals and organizations dispensing misleading nutritional matter.	
10. Speak out in speeches and public statements against nutrition misinformation, as-well as reiterating the facts concerning sugar.	
11. Counsel and exchange information with sugar-using industry groups.	
12. Organize two national mailings to doctors/1. Heart Disease, 2. Obesity.	
13. Gain equal time on TV by offering up medical experts to respond to inaccurate lay criticism, with major efforts to counter the misinformation dispensed during “Food Day” and the inaccuracies in the book, “Sugar Blues.”	
14. Publish “Sugar in the News,” a digest of what is being said about sugar concerning health, to determine reportorial balance.	
15. Regularly circulate mini-documentary radio tapes using prominent doctor and dentists as interviewees/200 stations.	
16. Encourage the use of the Assn. Washington office as a reliable source of nutrition information; recommend expert scientific sources.	
17. Work regularly with newspapers, wire services and magazines, providing background, short takes and filler, as well as assisting in the preparation of feature articles. Have requested balanced reporting.	
18. Maintain contact with key federal government health officials and regularly provide pertinent information regarding sugar and health.	
19. Attend conventions of professional dietitians, food technologists, home economists and various user-industry groups.	
RESULTS	
Measurement has been directed to short-term objectives. The Assn. has analyzed its results on the basis of’ the understanding it has developed with, its key publics (see objectives) with volume of publicity a secondary consideration. Emphasis has been placed on the program’s ability to stem, the flow of reckless commentary. With proper understanding the subject of sugar and health will inspire only nominal coverage.	
I. Medical Community: Organization of the Food & Nutrition Advisory Council and distribution of literature have won the attention of this public. Doctors countrywide have assisted in taking the facts of sugar to the public—speaking engagements, TV appearances, press interviews. The Assn. has been told its accuracy, willingness to discuss problems (dental caries) and funding of research has established credibility in the medical community.	
II. [Nutritional] Professionals: “Sugar in the Diet of Man” has become the primary sugar source document for dietitians and home economists, in that it provides the scientific facts to teach and convey the sugar story. Direct contact through the regional Information Program, mailings, convention sessions and participation in programs has established the Assn. as a ready source of substantiated information. Its stand against nutritional misinformation has won attention and respect: its position on basic nutrition corresponds to those of leading professional organizations.	
III. [Sugar-] Users: They now support Assn. recommended research, distribute. Assn. literature, seek counsel on nutritional matters and publicly defend sugar.	
IV. Media: Attacks on sugar in the media have diminished sharply, and those that appear tend to be far more objective. Positive commentary has helped establish reportorial balance. Some major magazines now maintain they tacitly endorse sugar. In challenging the networks, the Assn. has been able to gain valuable TV response time. Having gotten to know the national food-editors, the Assn. frequently has the opportunity to respond in print to local attacks SA has become a source of reliable information and a vehicle for introducing scientists to the media. Inquiries have increased markedly.	
V. Government: Two major announcements—one by the National Academy of Sciences and the other by the FDA’s GRAS (Generally Regarded As Safe) Review Committee have been highly supportive of sugar, making it unlikely that sugar will be subject to legislative restriction in coming, years.	

#### Crisis 1 – attacks on sugar’s safety

The primary crisis addressed by SA’s campaign was a “barrage of criticism in the media suggesting that public consumption of ever-increasing amounts of sugar was responsible for a far-ranging variety of health problems,” including that sugar was addictive [32]. Events inspiring negative coverage included the non-profit Center for Science in the Public Interest’s “Food Day,” a national event modeled after Earth Day to educate the public on the “nutritional, political and social drawbacks,” of a list of “terrible ten” foods, including sugar and sugary products such as Coca-Cola, breakfast cereal, and baby foods, [33] and a popular book, Sugar Blues which, among other things, compared sugar to heroin [34]. SA believed that consumer advocates had inspired California to propose “to tax sugar at the manufacturing level to fund a program of nutrition education for the state,” [35] to” tax soft drinks and its correlated advertising to raise $100 million to finance a socialized dental program,” [36] and Massachusetts to propose to “segregate sugar and sugar containing products in state retail outlets” [35].

SA was concerned that the media criticism was causing health opinion leaders, including physicians and dentists, nutritionists, and government health officials, to form negative attitudes toward sugar [32, 36] and affecting purchasing behaviors of individual consumers and food and beverage manufacturers [36]. SA was concerned about the agenda-setting power of the media relative to two upcoming safety reviews of sugar with regulatory implications sponsored by the FDA [32]. The first, to be conducted by the Federation of American Societies for Experimental Biology was part of a re-evaluation of the FDA’s previous decisions about of the safety of food substances deemed to be generally recognized as safe for consumption by the general population [37, 38]. The second, to be conducted by the National Academy of Sciences had been scheduled in 1974 to discuss concerns about the safety of saccharin, then, according to sugar executives, was “expanded to include sugar and a variety of health-related issues” and, “the proposed agenda with the subjects and suggested speakers [wa]s not favorable toward sugar” [39].

#### Crisis 2 – attacks on the sugar industry’s social legitimacy

SA’s Silver Anvil campaign also addressed a social responsibility crisis, with the strategy that it not “appear to be self-serving” [32]. In 1969, Abbott Laboratories, a major manufacturer cyclamates, a class of inexpensive artificial sweeteners, had publicly accused the sugar industry in the *New York Times* of inappropriately influencing the FDA to place restrictions on their products [40]. In December 1971, the Federal Trade Commission (FTC) accused the Sugar Association of “falsely advertising that eating sweets before meals helps people to reduce [weight],” [41] and ruled in November 1972 that “the ads’ weight reduction claims were false and misleading, and required SA to run corrective advertisements” [42]. Consumer advocates had begun questioning high level of sugars in pre-sweetened breakfast cereals marketed to children, characterizing the cereal products as “empty calories” [43]. In 1974, a spike in the price of sucrose caused the FTC to investigate the sugar industry for collusion [44]. In 1975, SA learned that the forthcoming book, *Sugar Blues*, would be providing “detail [on] how the sugar lobby has kept the lid on reports about its harmful effects” [34].

### SA’s corporate apologia

#### Crisis 1 -- product safety response – deny guilt and attack the accuser

SA’s primary crisis response strategy was counterattack, in which an organization claims that their accusers are at fault and denies guilt [16]. SA’s campaign materials described sugar’s critics as “food faddists,” “pseudoscientists,” “promoters,” [36] or “opportunists” who were motivated by selling books and magazines (e.g. Adele Davis, Rodale press, John Yudkin), ratings on TV or radio (e.g. Merv Griffin and Carlton Fredricks), selling alternative sweeteners (e.g. the health food industry), or selling vitamins to supplement a diet of highly-refined foods (Linus Pauling, Wilfred and Evan Shute, Emanuel Cheraskin) [45]. SA’s president characterized sugar’s critics as “capitalizing” on the public’s confusion over conflicting nutrition information “to promote questionable and often harmful products and services” [45]. Sugar was described as being a “natural target” for critics “because of its universal usage and visibility,” [32] compared to other potentially harmful foods and food additives. Accusations that sugar was addictive were described as unfair because “the psychology of sweetness works against sugar” [32]. SA’s president claimed that the sugar industry was disadvantaged due to the showmanship of the promoters, who targeted “television talk shows and unschooled feature writers who deal in sensation” [36]. Consumer advocates, who had been gaining media attention and legislative and regulatory traction against sugar, were characterized as being “well-meaning” but “mislead” by the “promotional onslaught” [32].

To address sugar’s role in dental caries, SA enacted a differentiation strategy, through which corporations acknowledge they are somewhat responsible, but argue that mitigating factors limit culpability [16]. SA’s president argued that it was “but one of many factors contributing to caries” and that it was “the frequency of [sugar] intake and the form in which it is taken [that plays] a far greater role than does over-all consumption” [35]. SA’s focus on the frequency and form of sugar consumption was an argument that the high amount of sucrose in in various products was not the important causative factor in dental caries, and therefore, if the FDA chose to limit the amount of sugar that food and beverage manufacturers could add to products, this action would not effectively reduce dental caries. SA also used a corrective action strategy, [16] in which the industry “acknowledge [d] sugar’s vulnerability in the area of dental caries and [pledged] to direct research to this problem” [32].

SA denied sugar’s guilt related to all other health effects using a typical appearance/reality dissociation, in which a corporation argues that there is a false perception of guilt, and the “true facts” of the case absolve the company (or its product) from wrongdoing [16]. SA’s campaign summary claimed that:

An independent survey of existing scientific data and literature search headed by Dr. F.J. Stare, chairman of Harvard’s Dept. of Nutrition, revealed sugar to be a safe food than in certain forms does contribute to dental caries. As well, it indicated that the desire for sweet is inherent and that U.S per-capita consumption had not changed in 50 years [32].

SA contrasted itself from sugar’s critics by describing itself as “[f]oster[ing] an independent Food & Nutrition Advisory Council (six doctors/two dentists)” to “organize existing scientific facts concerning sugar in a series of [six] papers” [32] on sugar’s role in nutrition, cardiovascular disease, obesity, diabetes, hypoglycemia, and dental caries [46]. In an effort to demonstrate scientific merit, the 88-page series, collectively titled “Sugar in the Diet of Man” (SITDOM) was published in *World Review of Nutrition and Dietetics* [46].

SITDOM’s introductory paper, prepared by Stare, titled “Sugar’s Role in Modern Nutrition,” [47] exemplifies an opinion/knowledge dissociation, in which a corporation denies guilt by arguing that critics’ claims are opinions [21]:

Sugar has been a familiar household food commodity in much of mankind for so long that it would seem unnecessary to evaluate its role in health, as we intend to do in this series of papers… [yet] [c]urrently, there are many voices reaching the public ear who make accusations against sugar as a food. These accusations vary from ascribing overweight and obesity to sugar, rather than to overeating, to much more serious charges, such as that sugar is an important cause of coronary heart disease. In fact, there are some who claim that sugar is not a food and not even a nutrient…This has resulted in a situation in which hypotheses, data which are incomplete, data from animal studies of questionable application to man, and data which are erroneous and simply ‘opinion’, are widely and repeatedly disseminated to the public as ‘facts’… The purpose of this series of papers is to balance hypotheses against known facts, and to put them into the perspective which will provide a useful evaluation of the true place of sugar in modern nutrition [47].

Building on this opinion/knowledge dissociation, the remaining SITDOM papers outline counter-arguments to claims in the media of sugar’s links to cardiovascular disease, [48] obesity, [49] diabetes, [50] hypoglycemia, [51] and dental caries [52].

Table 3 contains our thematic analysis of SITDOM’s counterarguments and reasoning as packaged for the media and government officials in a digest commissioned by SA [53]. These counterarguments are what Hearit describes as a “counter description,” which in a corporate apologia provides a narrative that attempts to alter the interpretation of an alleged offense [16].

**Table 3:** The Sugar Association’s Key Counterclaims to Anti-Sugar Claims in Media, with Supporting Reasoning [53]

Topic: Role of Sugar in Modern Nutrition	
Anti-Sugar Claim	SA Counterclaim
*Sugar consumption is rising in the United States*	*Sugar consumption has not changed over the last 50 years*
	*Reasons*: “Harvard’s Dr. Fredrick Stare reports that of the roughly 100lbs. of sugar consumed each year by an average American only some 80lbs. are actually eaten. This amount has scarcely changed in half a century. So claims that sugar consumption is rising in the United States are untrue. Industrial sugar use has grown, but direct consumption of sugar by the consumer has declined proportionately.”
*Sugar is not a nutrient or a food*	*Our primary nutrition need is energy. The bulk of our energy has to come from carbohydrates. Sugar is a compact, economical carbohydrate energy source.*
	*Reasons*: “Such charges [against sugar] are disturbing, for we use sugar not as a sweet extra but as a basic of our food supply. In the United States last year, slightly over 100 lbs. of sugar disappeared from the market for each living American, about 15 to 20% of our total calories. If sugar were removed from the market, all the calories needed to feed at least 36 million Americans would be lost.” “Worldwide, many nations—such as Great Britain, the Netherlands, Denmark, Australia and Israel—use as much or more sugar per capita than the United States. And consumption is rising in other lands, especially in developing nations. The U.N. reports an increased use of sugar unmatched by any other food for humans, predicting that by 1980 the world will consume some 186 billion lbs. a year.” “About 15 to 20% of a typical American’s calories come from sugar—about 12% for adults and 20% or more for teenagers. That sugar is seen by scientists as a compact source of energy, our primary nutrition need.” “Energy, Dr. Stare points out, can come from any of three food sources—proteins, fats or carbohydrates. Protein is expensive and scarce; it provides only about 15% of our calories. Excess fats may offer health hazards. So the bulk of our energy has to come from carbohydrates.” “So, Dr. Stare concludes that in the absence of a compact carbohydrate energy source, such as sugar, it could be hard to give youngsters enough calories without giving an unhealthful excess of fat.” “This energy compactness, coupled with high agricultural yield, makes sugar a solution to some world hunger problems. No other major crop yields so many calories from so little soil—about 1 million calories from an eighth of an acre. To produce so much energy, potatoes take four times as much land, beef 125 times. And energy, fertilizer and refining costs for sugar are relatively low.”
*Sugar is an unhealthy carbohydrate*	*Sugar is no different than any other carbohydrate*
	*Reasons*: “(Many people are unaware that virtually all carbohydrates in our food are either sugars, or, as in the case of starches, are chains of sugars. Sucrose, the scientific name for table sugar, is one of the most common sugars found in plant foods.)” “The energy we get from sugar is scientifically no different from that in any other food. But most other carbohydrate-containing foods, such as fruits and vegetables, have much bulk with few calories.”
*Sugar is empty calories*	*Americans would not consume a healthy balanced diet full of vitamins, minerals without sugar to make foods palatable*
	*Reasons*: “Though they carry with them no vitamins, minerals or protein, Dr. Stare notes that sugar is rarely eaten alone. And often it makes more palatable many foods such as cereals and fruits that supply other valuable nutrients.”
Topic: Sugar and Cardiovascular Disease	
Anti-sugar Claim	SA Counterclaim
*Coronary Heart Disease (CHD) deaths are higher in countries where more sugar is eaten*	*Some countries with high CHD mortality do not have high sugar consumption rates*
	*Reasons*: “Finland and Sweden use similar amounts of sugar, but the Finns have far more CHD. And a number of nations—such as Venezuela, Cuba, Colombia, Costa Rica and Honduras—consume much sugar but have a low incidence of CHD.”
*Increases in heart disease-related deaths can be accounted for by rising sugar use*	*Sugar consumption has not changed in the U.S. over the last 50 years*
	*Reasons*: “Dr. Grande finds that the increase of CHD deaths in the United States during the last 50 years occurred while sugar use stayed constant.”
*Emerging evidence links sugar to CHD*	*Dr. John Yudkin’s work is the primary source linking sugar to CHD, and his work has been discredited*
	*Reasons*: “Dr. John Yudkin (author of nearly all the medical papers linking sugar to CHD) says that 20 of his heart patients ate more sugar than the usual. But nine other studies controvert this finding. In two of them the heart patients ate less. In one, Canadian veterans with CHD averaged 47 g of sugar a day; but a matched group of healthy veterans ate 65 g!”
*Sugar increases blood lipids, such as cholesterol and triglycerides, indicating a higher risk of CHD*	*Sugar does not increase blood lipids*
	*Reasons*: “Reviewing scores of studies—in some of which sugar was the source of 75% or more of calories—Dr. Grande can document no such effect. The only indicators have been a few experimental studies of people who already had high levels of blood fats and who were fed huge amounts of sugar in a special dietary far different from any normal eating pattern. Such anomalies can hardly explain a disease so common that it is our worst killer.” “When large amounts of carbohydrates replace large amounts of fat in the diet, there is a rise in some people of certain fats in the blood (triglycerides). But this rise is temporary. And people who regularly eat much carbohydrate prove to have lower levels of such fats in their blood.”
*Current sugar consumption levels are a causative factor in CHD*	*Sugar consumed at current levels does not cause CHD*
	*Reasons*: “To conclude,” says Dr. Grande, “the evidence available does not support the view that sugar, in the amounts present in diets such as those consumed in this country, is a causative factor in the development of CHD.”
Topic: Sugar and Obesity	
Anti-Sugar Claim	SA Counterclaim
*Sugar causes obesity*	*Sugar does not cause obesity*
	*Reasons*: “Three University of Pittsburgh teachers of medicine—Drs. Thaddeus Danowski, Sean Nolan and Thorsten Stephan—review the evidence and conclude it does not.” “The stage seems to be set for obesity when some of us are either born with more of the storage cells that hold body fat or increase them by overfeeding in infancy. Even if one loses weight, these fat cells are not destroyed.” “The fat cells can be filled or emptied depending on how many calories of energy we get in food and how many we burn with exercise. It does not matter which food the calories come from. Any excess is stored as fat.” “Most such excesses seem small and steady. The experts conclude that often they result from learning to deal with emotional stress by eating more. Conversely, excesses may result from declines in activity, as with aging.” “Two other possibilities are suggested to account for individual tendencies to fatten. One is that while some of us stir and bustle more others are more economical of energy. Also, some of us may use fuel more efficiently, like cars that get more miles from a gallon; In either case tiny excesses can add up. Even a 1-per-cent-more efficient use of fuel could mean a gain of 40 lbs. over 20 years!”
*Diets restricting sugar will lead to weight loss*	*Diets restricting sugar will not lead to weight loss*
	*Reasons*: “Because only total calories matter, it does not help weight control to restrict only starches or sugars. Like protein, they provide some four calories to the gram. Some restriction of fats may be useful, however, for they have nine calories to a gram.” “Unhappily, most people who want to reduce tend to limit their diets to a few foods and eliminate many others. This leads to boredom and failure and to poor nutrition.” “For example, low-carbohydrate diets are pointless and may be hazardous. And when such diets emphasize meats they prove to be very high in fat. Although they seem to work for some people, they can cause nausea, loss of appetite, and the loss of body fluids (dehydration). The AMA has warned doctors of their medical dangers.” “Even skipping meals is self-defeating. For the body tends to deposit more fat with only one or two meals a day then with several, although the caloric intake is the same.” “The most successful programs of caloric restriction,” say the authors, “deviate least from ordinary eating... Any excess of calories, be it from food, alcohol or inactivity, is inevitably stored as body fat. It is this excess of calories, and not the type or amount of sugars, starches, protein or fat in foods... that results in obesity.”
Topic: Sugar and Diabetes	
Anti-sugar Claim	SA Counterclaim
*Diabetics should restrict their carbohydrate intake*	*Diabetics do not need to restrict their carbohydrate intake*
	*Reasons*: “In 1971 the American Diabetes Association surprised many with a statement that: “There no longer appears to be any need to restrict disproportionately the intake of carbohydrates in the diet of most diabetic patients.”” “Explaining this statement, Drs. Edwin Bierman (who chaired the committee that wrote it) and Ralph Nelson note that today patients rarely die of the direct effect of diabetes. They are lost to ills of the heart, blood vessels and kidneys, all of which may be related to high-fat diets. And a diet low in carbohydrates, such as the traditional diabetic diet, is high in fat. As the ADA statement observes: “A liberalized carbohydrate intake…will necessarily be associated with a decrease in dietary fat and cholesterol.””
*Carbohydrates are harmful to diabetics*	*Carbohydrates are not harmful to diabetics, and may help diabetics live longer*
	*Reasons*: “Newer studies indicate that carbohydrates seem not only to stimulate the body to produce insulin, but also to trigger enzymes that put sugars in the blood to work. Experiments using diets up to 80% sugar actually improved the glucose (blood sugar) tolerance of both normal people and diabetics.” “Bierman and Nelson cite evidence that total calorie control is the main nutritional need of diabetics. If there is such control, diabetics can eat as much carbohydrate as the normal American—45% or more of the calories they consume.” “Will more carbohydrate and less fat help diabetics live longer? No one is yet sure. But in Japan, where diabetics eat more carbohydrates than their Western counterparts, they show less glucose intolerance and suffer less the complications of gangrene, while other functional disturbances are similar for both groups.” “Regulating the diabetic’s diet is still important. It must be adjusted to medication, activity and, above all, to calorie needs. And sudden surges of blood sugar (as can be caused by large amounts of sweets) must still be avoided. For the obese diabetic total calorie restriction (calories from all sources, not just sugar) is not relaxed.”
*Sugar causes diabetes*	*Excess sugar consumption does not cause diabetes*
	*Reasons*: “There is no evidence,” the researchers emphasize, “that excessive consumption of sugar causes diabetes.”
Topic: Sugar and Hypoglycemia	
Anti-Sugar Claim	SA Counterclaim
*Hypoglycemia is a widespread condition*	*The prevalence of hypoglycemia is overstated*
	*Reasons*: “Low blood sugar” (hypoglycemia) is a new way to explain many discomforts, especially emotional ones. “We may find it easier,” say the experts who studied the data, “to say, ‘My blood sugar is low,’ rather than, ‘I can’t cope.’”
*All cases of hypoglycemia require treatment*	*Not all cases of hypoglycemia require treatment*
	*Reasons*: “But contrary to popular belief, not all of those people who show somewhat low blood sugar levels in tests are ill and need special care. Indeed, Pittsburgh’s Drs. Danowski, Nolan and Stephan find that 20 to 30% of normal people can show such readings in glucose tolerance tests.” “Low blood sugar is simply a sign—one that should be checked to eliminate the possibility of serious underlying disease. This is especially true if the hypoglycemia is moderate or marked.” “In most cases of mild hypoglycemia (blood sugars of 45 to 60 mg per cent) there is nothing more than a variation of normal. But even such patients should be followed for a time.” "In moderate hypoglycemia, however (30 to 45 mg. per cent), the low blood sugar may signify a prediabetic state. In a Public Health Service survey, within 10 years over 50% of those with moderate hypoglycemia progressed to actual diabetes. All cases showing very low blood sugars (zero to 30 mg. per cent) are unusual. And blood sugar at this level may be the indicator of poorly controlled diabetes mellitus or of some rare and serious diseases, such as disorders of the nervous system, the liver, certain glands and the life."
*Hypoglycemia causes psychological disorders*	*Hypoglycemia does not cause psychological disorders*
	*Reasons*: “These slightly low blood sugars are not, as some claim, the cause of psychological disorders.”
*Hypoglycemia is caused by too consuming much sugar*	*Hypoglycemia is not caused by consuming too much sugar*
	*Reasons*: “[Low blood sugars are not] the result, as some popular writers insist, of too much carbohydrate, especially sugar, in the diet.” “Whatever the reason for low blood sugar, it is the result of either an individual quirk or a disease state, not of eating starches and sugars. A diagnosis of “low blood sugar,” the authors’ studies of research suggest, is not a diagnosis at all. In many cases it is meaningless. In some others it is a signal to look for what could be an underlying disease.”
Topic: Sugar and Dental Caries	
Anti-sugar Claim	SA Counterclaim
*Sugar is the cause of tooth decay*	*Sugar only causes decay if it is consumed between meals and in sticky form*
	*Reasons*: “the facts [supporting the claim that sugar causes tooth decay] are not so simple” “For example, in a five-year study at a Swedish mental institution patients were divided into seven groups. The first group got no sugar but still developed some cavities. Two other groups received sweet drinks at meals but had no increase in decay.” “Four other groups received between-meal sugars—as beverages, chocolate, caramels or toffee. The beverages and chocolate had no significant effect on decay, but the sticky candies did increase decay markedly when taken between meals.” “From this and many related studies, Drs. Sidney Finn and Robert Glass conclude there is not a one-to-one relationship between total sugar consumption and decay. But there is strong association between cavities and between-meal eating. It seems to be long and frequent exposure to certain foods in certain forms that does the damage.”
*Foods should be ranked based on their cavity-causing potential*	*Foods cannot be ranked by their cavity-causing potential*
	*Reasons*: “Which foods? Nearly all sugars tested cause about equal decay. “Natural” sugars, such as honey, are as decay-producing as table sugar. The common sugars of grains, fruits, vegetables and even milk may produce decay. And cooked starches can be as troublesome. For two years 979 children were given as much unsweetened or presweetened cereal as they wanted; regardless of the kinds or amounts of cereal consumed, the amount of decay was similar.”
*Dental caries should be controlled by reducing sugar consumption*	*Reducing sugar consumption is not practical. There are other effective means of dental caries control.*
	*Reasons*: “Why? Drs. Finn and Glass describe decay as an infectious disease. For mouth bacteria, which flourish on carbohydrates, produce an acid waste that eats into tooth enamel. Good hygiene helps to reduce tooth-adhering plaque and bacteria harbored by the plaque but never eliminates them entirely.” “Many other factors enter into decay—heredity, the shape, alignment and hardness of teeth, the chemistry of the saliva and so on. But there are few of these factors we can control. We cannot keep our mouths bacteria-free or go without all the foods on which the bacteria can feed.” "We can practice good dental hygiene, including regular visits to the family dentist. We can avoid sticky foods containing sugar, particularly between meals and at bedtime, and opt for sweets in liquid or quick-dissolving forms. But above all, we can add fluorides to school and community water supplies to harden young teeth early. It is estimated that for about 25 cents per person annually community water supplies can be fluoridated and some 60% of dental decay (and hence dental bills) can be eliminated."
Topic: Other	
Anti-sugar Claim	SA Counterclaim
*Sugar has been linked to health problems beyond obesity, heart disease, diabetes, and dental caries, including addiction.*	*Claims that sugar is addictive are not credible*
	*Reasons*: “Many other human ills have been charged to sugar. But in these evidence is either non-existent or so slight that the scientific reviewers saw no reason for further study.”
*The FDA should ban sugar*	*Sugar is safe for the general public at current consumption levels*
	*Reasons*: “In 1973 a team of noted scientists all of whom were either physicians or dentists—aware both of such threats and of sugar’s mounting importance as a food—began to study what was actually known about sugar’s effects on health. Working independently, at medical centers and universities across the nation, they combed the archives of scientific research. This is a summary of the six recently published papers in which they report their findings (World Review of Nutrition and Dietetics, Vol. 22, pp. 237–326, 1975).” “In general they conclude that sugar, like any other widely used food, is harmless when eaten in reasonable amounts.” “Considering all that science has learned of sugar and health, Dr. Stare sums up: "Sugar, a pure carbohydrate, is an important nutrient and food in the U.S. diet when used in moderation... Studies of actual intake suggest that the percentage of calories taken as sugar are higher during the growing and adolescent years when energy demands are high, and lower during adult and later years... There being no valid evidence to the contrary, this rate of intake (between 10 and 30% of total calories, with the average at 15 to 20%) may be considered moderate.” “Discussing the conclusions of the investigating team, Dr. Stare concludes, simply "At these levels sugar contributes to good nutrition, to the enjoyment of our meals; and it is safe.”

#### Crisis 2 – social responsibility response--admit guilt and claim reforms

SA used apology and corrective action [16] as its response to its social responsibility crisis. Corporate apologies typically utilize act/essence dissociations, which argue that while an act was committed, it was not representative of the essential nature of the organization [16]. SA sought to dissociate itself from its own false and misleading advertisements that eating sweets before meals helped people lose weight by “drop[ping] all Assn. advertising” and “mov[ing] to a program of public information and education” [32]. Like many corporations concerned with lawsuits, [21] SA did not actually accept responsibility for its advertising behavior, instead characterizing its new focus on public information and education as necessary because “a nutritional message [disseminated via industry advertising] would be seen as self-serving” [32].

As part of further efforts to demonstrate social responsibility, SA stated that the industry would “[l]et qualified medical experts speak for the industry, regardless of potential negatives” [32] and “[s]eek media objectivity and balance, not necessarily extensive coverage” [32]. It claimed that in contrast to promoters, opportunists, and pseudoscientists, SA would also “[p]romote an understanding of sound basic nutrition” [32] and “[s]tand up publicly against the purveyors of nutrition misinformation” [32] as a way to reduce public confusion about conflicting nutrition information.

### Ethics of SA’s corporate apologia

Hearit’s Corporate Apologia theory [16] provides standards by which to ethically judge the form of corporate crisis communication (Table 1), which encompass six categories.

#### Truthfulness

One measure of a corporate apologia’s truthfulness is whether it omits information that, when revealed, would fundamentally change how others view an apologists’ actions [16]. SA did not disclose to PRSA or to the public that SITDOM, rather than being a collection of existing scientific facts gathered by independent scientists, was actually the culmination of a 5-year effort to exonerate sugar that was funded by the sugar industry and directed by public relations agencies.

#### SA’s development of sugar in the diet of man

The origin of SITDOM, the scientific substantiation of SA’s corporate apologia, was the report “COPING WITH NEW DANGERS: An Action Plan for Sugar Information in the Face of Mounting Criticism, Public Relations Proposals for 1971-1972” prepared by the public relations firm Ruder and Finn for SA in 1971 while engaged by the sugar industry to run “an aggressive, single-track effort to spell out why cyclamates were neither safe nor necessary” [54]. Ruder and Finn had noticed an upsurge of “accusations [about sugar’s negative health effects] that [we]re appearing in professional medical publications – science magazines – consumer magazines and newspapers –books – on radio and television” [54].

Ruder and Finn outlined a strategy called “Taking the Offensive,” which SA ultimately adopted. To succeed, it advised SA it would need to:

- convincingly state sugar’s value as a food.
- point out, in appropriate media, the research-based truth about sugar in relation to health and heart disease.
- anticipate further criticisms of sugar, whenever possible.
- demonstrate that the sugar industry is aware of its responsibilities in our society – that it does not intend to by-pass any real problems connected with sugar consumption of such problems actually exist—that it is willing to answer the questions raised by consumerism or the scientific community [54].

Ruder and Finn noted that achieving these goals would be “a large order” [54].

In 1971, sales of sugar had not yet been affected by media criticism, however Ruder and Finn felt action was urgently needed because of a high likelihood that “money [wa]s being spent to destroy the popular acceptance of sugar” on the part of what it described as the “anti-sugar front.” The firm believed “perhaps the most disturbing aspect of the new situation” was the emergence of “a relatively large amount of research – good and bad – that sugar critics can draw on. Collectively, it represents the Bible of the anti-sugar forces” [54].

Ruder and Finn counseled SA “to make sugar’s case” by “advancing a positive statement about sugar in relation to health problems and nutrition” to “more people, in more media” [54]. They advised that the positive statement should “provide criteria for judging the challenges to sugar” because “the general public, as well as opinion-making segments of the public such as doctors, teachers, science writers and educators, would never be fully knowledgeable about sugar” and could not be expected “to apply critical yardsticks to the attacks that sugar is sustaining in the press and on the air” [54]. They advised that the statement should “present true facts, and talk about them in light of what consumers and specialists want to know, rather than what we prefer to tell them” [54]. The goal, according to Ruder and Finn, was for the industry to be seen as “intelligent reporters, not propagandists” and as serving the public by helping it “make its best judgments” [54].

To develop a positive statement about sugar, Ruder and Finn told SA it would need an “increased input of usable information” [54]. The firm noted that “one of massive difficulties in combatting sugar’s critics, and in demonstrating the safety of sugar in normal consumption, is the lack of information on many key points” [54]. Because “much of this research is not easily available – or is simply unfamiliar—or ha[d] never been attempted,” SA would need to make available data produced by the industry in the past, as well as sponsor new research [54]. Ruder and Finn provided a preliminary list of “some of the soft spots in discussing sugar and health, some of the things *we don’t know well enough* [emphasis in original]” that would need to be addressed:

- Validity of [rising] per capita sugar consumption figures since 1840
- Responsible criticism of the claims that Yemenite Jews in Israel suffer from disease caused by increased sugar intake
- Dependable correlations of the incidence of heart disease, diabetes and hypertension with the consumption of sugar during the past 50 years
- The usefulness of artificial sweeteners and artificially-sweetened foods in weight control
- Comparisons of the average diet in the U.S. with foreign diets and the incidence of heart disease
- The facts on which Finland and Sweden acted to recommend reduced consumption of sugar – and the results
- The meaning of the USDA studies at the Carbohydrate Nutrition Laboratory at Beltsville, Maryland
- The amount of sugar added to baby foods, and the effects of such addition
- An interpretive study of the effects of a drop in the U.S. intake of carbohydrate but an increase in the amount of sugar vs. starch
- Correlation of U.S. sugar intake with mortality rates, stature, weight and longevity figures for at least 50 years
- The acceptance of Yudkin’s claims [that sugar contributed to heart disease] by American and foreign cardiologists and nutritionists
- The incidence of dental caries compared to sugar intake in the U.S. and foreign countries
- The pluses (if any) and the minuses in the use of unrefined sugars and molasses for human consumption
- Advance news of further research now being conducted on relation of sugar to health sources [54].

To collect the data necessary to support a positive statement about sugar, Ruder and Finn urged SA to consider utilizing the International Sugar Research Foundation (ISRF), an allied trade organization, “with its science orientation and contacts with researchers” [54]. It also advised SA to consider forming its own scientific advisory board or using an outside science consultant. Another possibility was to employ “a science aid on the Sugar [Association] staff, helped by an assistant to probe the literature in fields of our interest” [54]. Additionally, the firm urged that:

However this need is supplied, it must be accepted that information input is a continuous activity. It cannot be satisfied occasionally through reading newspaper clippings or checking some of the science press. It requires trained personnel with scientific background, and a sense of the appropriateness of certain facts, figures and studies for the needs of [SA] [54].

To disseminate the positive statement in various media channels, Ruder and Finn advised SA to identify “eminent spokesmen” because:

What the sugar industry tells the public about sugar must be considered self-serving. What we say tends to be questioned. What eminent scientists, researchers, physicians, and nutritionists say about sugar carries weight in our science-minded society. What they say tends to be believed [54].

Unlike the past when the sugar industry relied on “one or two well-known nutritionists,” Ruder and Finn felt SA’s new spokesmen:

must include specialists in the fields of cardio-vascular disease, bio-chemistry, diabetes, overweight and nutrition. They may be researchers, practicing physicians, teachers at medical centers, or health authorities. They must be willing to express their ideas and opinions about the effects of sugar and health – and their ideas and opinions must on the whole be favorable, or at least non-critical of sugar [54].

As of March 1971, Ruder and Finn had already begun “a new search for such authorities” and believed they would “be able to enlist a number of cooperating scientists and writers whose work entitles them to professional respect” [54]. Finally, to “focus [SA’s] advertising and public relations activities in the most effective channels,” Ruder and Finn recommended conducting a public opinion poll. If favorable, SA would “be able to use [the results] in its public relations materials.” If not, SA would “know in what direction to change its priorities” [54].

Table 4 lists sugar industry sponsored activities initiated immediately after SA received Ruder and Finn’s 1971 report, which led to the publication of purportedly independent SITDOM series in 1975. The largest sugar manufacturer at the time, Amstar, took the lead in recruiting a Food and Nutrition Advisory Council (FNAC) [65] to “help the Company determine the validity of [the increasing attacks on sugar] and thereby be in a stronger position to refute those that are incorrect and take corrective action when needed” [58]. Amstar also had a leadership role in ISRF [66] and was a founding member of another food industry trade group the Nutrition Foundation (NF) [67]. A key member of Amstar’s FNAC was Fredrick Stare, [68] chairman of Harvard’s Department of Nutrition, who already had a history of advising ISRF (beginning in 1965 when it was known as the Sugar Research Foundation (SRF)), [69] and had connections to NF [70]. Stare had advised SRF on research projects that questioned the effectiveness of artificial sweeteners [71] and had been the senior author of a sugar industry-commissioned review published in the *New England Journal of Medicine* in 1967 [72, 73] that accentuated the uncertainty of emerging evidence linking coronary heart disease to sucrose while overstating the certainty of the evidence linking CHD to consuming saturated fat [74].

**Table 4:** Sugar Industry Activities Leading to the Sugar Association’s 1975 Publication “Sugar in the Diet of Man”

Dates	Sugar Industry Organization	Key Actions
Mar. 1971	Sugar Information, Inc. / Amstar	• Received report title “Coping with new dangers: an Action plan for Sugar Information in the face of mounting criticism” from public relations firm Ruder and Finn, Inc. [54]
Feb. / Mar. 1971	Amstar	• Organized the Amstar Food and Nutrition Advisory Council (FNAC) [55]
March 1972	International Sugar Research Foundation	• Held symposium: “Sugar and Human Health” [56]: o Topic 1 – “Sugar in Nutrition” o Topic 2 – “Atheroma, Is Sugar Involved?”
Sept. 1972	International Sugar Research Foundation	• Held symposium “Sugar, Growth and Development, Energy, Sucrose and a Balanced Diet.” [57]
Nov. 1972	Amstar / Nutrition Foundation	• Held symposium: “Sugar’s in Nutrition” [58, 59]
1973	The Sugar Association	• Organized its own FNAC [53]
Aug. 1973	Marabou	• Held symposium: “The Role of Sugar in Modern Nutrition” [60]
Sept.1973	International Sugar Research Foundation	• Held symposium: “A Research Profile of Sugar as a Food” [61]
Dec. 1973	The Sugar Association	• Held its first FNAC conference to collate “Sugar in the Diet of Man” [47]
Jan. 1974	The Sugar Association	• Held its second conference to collate “Sugar in the Diet of Man” [47]
Mar. 1974	International Sugar Research Foundation	• Held symposium: o “Is the Risk of Becoming Diabetic Affected by Sugar Consumption?” [62]
Sept.1974	International Sugar Research Foundation	• Held symposium: o “Dental Caries: A review of current research on the prevention of cavities Part 1” [63]
1974	Nutrition Foundation	• “Sugar’s in Nutrition” published as the first monograph in a new series [59] ▪ Based on November 1972 symposium.
Mar. 1975	International Sugar Research Foundation	• Held symposium: “Dental Caries: A review of current research on the prevention of cavities Part 2” [63]
1975	International Sugar Research Foundation	• Published a commissioned a review: “The role of sucrose in foods, A comprehensive review of 30 years of research” [64]
1975	The Sugar Association	• “Sugar in the Diet of Man” published without disclosing sponsorship [46]
1975	Amstar	• Re-directed its FNAC to focus on company specific issues in 1975 after executives had determined that SA’s FNAC was “well organized and competent.” [65]

Between March 1972 and March 1975 SA, ISRF, NF, and Marabou, a Swedish chocolate company, would host eight symposiums that examined the positive role of sugar in nutrition and questioned its role in heart disease, diabetes, and dental caries, several of them led by Stare [56, 57, 60–63]. In 1973, SA and ISRF together contracted with National Analysts, then a division of Booz Allen Hamilton, to conduct an opinion poll of physicians, dentists, nutritionists, dietitians, government officials, and consumers to help them tailor their positive messaging about sugar for different audiences [75]. In December 1973 and January 1974, SA held two conferences, again led by Stare, to synthesize the data gathered by the industry to defend sugar into the six-part SITDOM series [53]. Stare was also the editor of the *World Review of Nutrition and Dietetics*, which published the series [76]. SA ordered 25,000 reprints of SITDOM, [36] commissioned a digest of its key points, [53] and packaged it together with a press release [77] which it disseminated to the media and to government health officials [78]. Authors of SITDOM would go on to become the first members of SA’s own FNAC [32]. In 1975, after Amstar executives determined that SA’s FNAC was “well organized and competent,” they re-directed Amstar’s FNAC to focus on company specific issues" [65].

SA’s counterclaims (Table 3) align with Ruder and Finn’s advice to convincingly state sugar’s value as a food, and to address sugar’s weak spots related to rising consumption levels, heart disease, obesity, diabetes, and dental caries based on “true facts” [54]. However, these “true facts” omitted key information related to the overall truthfulness of the campaign. For example, it was true that sucrose consumption had not changed over the last 50 years. However, SA knew that the per capita consumption of all nutritive sweeteners had indeed risen, as indicated in a speech given by SA’s President to an industry meeting in in 1975:

Now let’s go back and review the whole nutritive sweetener market for the past 40 years. In 1935 the sucrose share of the per-capita figure was 99 lbs., with corn sweeteners at 7 lbs.—a total of 106 lbs. In 1974 the sucrose share of the total nutritive sweetener market was 97 lbs., corn a healthy 24 lbs. and honey, etc., about 2 lbs., making a total nutritive market of about 123 lbs. per capita [36].

Similarly, SA’s claim that sucrose makes healthy foods more palatable ignored the fact that it also makes unhealthy foods more palatable. Multiple reviews cited in SITDOM had found no evidence linking sugar to heart disease, however these reviews had been funded by the sugar industry [72, 73, 79, 80].

#### Sincerity

To deliver a sincere corporate apologia, an organization must show evidence of a true desire to reconcile with offended stakeholders rather than wanting only to escape negative media attention [16]. SA’s support for dental research gave the appearance that the industry sought to address the problem of dental caries. However, SA’s decision to fund dental research was made in response to results obtained from National Analysts’ opinion poll [75]. SA claimed in its application for the PRSA award that its survey “suggested that support for sugar was stronger than anticipated and that sugar’s primary publics would be receptive to the scientific facts,” [32] but did not include the survey results. These data actually indicated that “Dentists display[ed] more consistently negative attitudes toward sugar than all other opinion groups included” and that “[t]he official position of the American Dental Association [ADA] is aggressively anti-sugar” [75]. National Analysts told SA that dental research was urgent because, “Dentists [we]re cited by almost half (46%) of all consumers as their source of information about sugar and health – a larger proportion than mention any other single source.” National Analysts advised that funding dental research, together with SA’s message about the form and frequency of consumption being more important in dental caries than the overall amount of sugar consumed, might “lessen concern about sugar and dental health and possibly win approval of the A.D.A” [75]. This implies that the purpose of sugar industry funded dental research was to reduce dentists’ policy engagement around sugar, rather than a good faith effort to prevent dental caries.

SA’s efforts to repent for its past false and misleading advertising practices by “promot[ing] an understanding of sound basic nutrition” [32] in the public’s best interest were also insincere. For example, SA told PRSA it had “Produced a 26-minute film (“Nutrition Is”) on basic nutrition for general distribution, that does not discuss sugar [emphasis in original],” [32] which gave the appearance that SA’s efforts were not economically motivated. However, SA did not disclose to PRSA the content of the teachers’ guides and lesson plans that accompanied the film, which contained SA’s counterarguments against the negative media claims about sugar [81].

SA’s efforts to “stand up publicly against the purveyors of nutrition information” [32] were less about protecting the public from misinformation than about disseminating industry messages. National Analysts advised SA that “Sponsorship of [its] informational program should be divorced, to the maximum extent possible, from the sugar industry. The industry, and its promotional/ research arms, are viewed with great skepticism by health opinion leaders” [75]. Internal documents demonstrate that SA supported “university and professional information campaigns against individuals and organizations dispensing misleading nutritional matter” because of their pro-sugar stance [78]. Representatives from these programs presented at conferences alongside SA leaders, and provided third-party credibility for the industry’s characterization of sugar’s critics as pseudoscientists, and of evidence of sugar’s adverse effects as nutrition misinformation [45].

## Discussion

The Sugar Association and Carl Byoir and Associates’ 1976 Silver Anvil award-winning public relations campaign successfully countered a large volume of media criticism linking sugar to diabetes, heart disease, obesity, hypoglycemia, dental caries, and addiction using a corporate apologia. This finding extends our knowledge of SA’s campaign beyond previous accounts [12, 13] by demonstrating that SA used rhetorical strategies to defend sugar and the sugar industry that were developed and supported by public relations agencies and common in addressing corporate crisis communications. In areas that the sugar industry believed it could deny guilt (sugar’s role in causing heart disease, diabetes, obesity, and addiction), SA used counterattack to re-frame sugar as a food necessary to achieve a balanced diet, and to re-position the sugar industry as a source of “true facts [sic]” about sugar, nutrition, and health. When denying guilt was unlikely to be successful (sugar’s role in dental caries), SA used a differentiation strategy that protected sales of sugar by prescribing reductions in the frequency of sugar consumption not the overall amount. SA used apology and corrective action intended to distance the organization from its past false and misleading advertising practices by sponsoring nutrition information programs, and from sugar’s role in dental caries by funding research on dental caries interventions. Underlying these rhetorical strategies was the industry’s counter-premise that sugar restrictions and those promoting them would be ineffective and cause harm.

SA’s counterattack relied on partial truths. Sugar makes all foods more palatable, which led the industry to claim that sugar was necessary to make healthy foods more palatable. Producers of artificial sweeteners and authors of popular nutrition books, among others, benefitted financially from criticizing sugar, which allowed SA to question the motivation of its critics and to suggest that consumer advocates had been taken advantage of by opportunists or food faddists. Some companies marketed products of questionable safety with unsubstantiated nutrition information, which SA used to suggest that all anti-sugar nutrition information was pseudoscience. Similarly, while some scientists supported the claim that there was no evidence linking sugar to chronic disease, SA did not disclose that they were sugar-industry funded scientists.

SA relied on a network of industry organizations to support its crisis communications. Working together with ISRF, NF, and Marabou, SA engaged in a form of information laundering. Like the tobacco industry did beginning in the 1950s in response to growing public awareness of the dangers of smoking accompanied by demands for regulation, advertising restrictions and health warnings, [82, 83] the sugar industry, working with public relations agencies, orchestrated the production of seemingly independent scientific articles that gave the appearance that industry claims about sugar were supported by evidence. Maintaining the appearance of independence was important to the credibility of the SA’s claims because the 1970s social environment was generally suspicious of corporate actions, and would likely have been skeptical of science sponsored by the sugar industry. SA’s scientific credibility was bolstered by SA’s efforts to “educate” the public on nutrition and protect it from “nutrition misinformation” from sources that were raising concerns about sugar in the diet [32]. This study adds to a growing literature describing historical and contemporary efforts by the sugary food and beverage industry to maintain a social, policy, and regulatory environment favorable to consuming unhealthy levels of added sugars through aggressive lobbying of regulators and legislators, [84, 85] the co-opting of domestic and international nutrition experts, deceptive marketing to children, targeting of minorities and emerging economies, [86] influencing research agendas, [87] and undisclosed conflicts of interest, [74] among others.

Scholars studying industries that seek to influence regulation and legislation have developed taxonomies for categorizing their tactics and arguments, drawing from business literature, as a means to understand broad patterns of political activity [6,7,8,9]. These researchers seek to aid public health advocates and policymakers in understanding corporate political strategies and prepare them to accurately assess and counter industry claims. Our case study adds to this work by demonstrating that the crisis communication literature is a useful lens through which to understand SA claims about nutrition and health. This study also has implications for the growing interest in countermarketing campaigns as interventions to reduce the demand for unhealthy foods and beverages. [88] Countermarketing campaigns use health communications strategies that expose the motives of producers of unhealthy products and portray their marketing behaviors as outside the boundaries of appropriate corporate behavior. Countermarketing can be used to reach individual consumers, regulators, influencers of public policy, journalists, investors, and corporate executives. Our findings on the unethical nature of SA’s corporate apologia contributes to our knowledge of questionable corporate behavior that could be incorporated into advertising campaigns. Our findings on the sugar industry’s messaging strategies could inform the development of counter-messaging for a public relations campaign.

SA created its Silver Anvil-winning campaign at a time when corporate apologia as a phenomenon were on the rise [16]. The success of social movements of the 1960s, coupled with the increasing availability of information, meant that corporations in general faced unfavorable media coverage and unprecedented legislative threats to their existing operations [89]. Between the 1960s and early 1980s, 26 major new health, safety, and environmental policies addressed tobacco and alcohol abuse, pollution, and food quality. [90] Although businesses had always been concerned with public opinion, this new social environment led to new developments in corporate communications [89]. Organizational discourse became a corporate resource.

The sugar industry spent years, supported by public relations agencies and a global industry network, developing and disseminating its defense of sugar. This defense was designed to prevent regulation and tailored for specific target audiences. SA recognized the importance of translating complex and nuanced scientific information about sugar, nutrition, and health for the media, health professionals, government officials, and the general public in ways that promoted a narrative that protected sugar from regulation and legislation. SA viewed key opinion leaders and consumers as susceptible to partial truths and to insincere efforts to demonstrate corporate social responsibility, [54] and exploited this weakness to advance corporate interests. SA did not accurately represent its campaign to PRSA when it applied for the Silver Anvil award. Its submission was the industry’s corporate apologia in its final form with key activities and decision-making related to the industry’s strategy omitted. This lack of disclosure has implications for public relations practice and education, as Silver Anvil submissions are central to public relations pedagogy [91].

This research has limitations. Our analysis of SA’s Silver Anvil award winning public relations campaign is a single case study, reviewing the history of a single trade association at a time when the industry faced immense political threat. The documents could not be verified using personal interviews due to the historical nature of the events. Our analysis was limited to an examination of SA’s crisis response strategies and we did not examine instrumental tactics or measures of public influence for SA’s Silver Anvil campaign.

Although SA’s public relations practices have evolved over the last 50 years, SA’s 2018 rhetorical strategies remain similar to those used in 1976. SA’s CEO, Courtney Gaine, has described the landmark decision by Secretaries of DHHS/USDA to endorse a 10% daily limit on added sugars consumption as “ineffective at best, and possibly destructive at worst” [92] because “sugar makes many healthful foods palatable, which helps contribute to increased intakes of many essential vitamins and minerals needed to maintain good health” [92]. These claims extend the 1970s premise that regulations restricting sugar consumption are ineffective and even harmful. In 2018 SA continued to characterize sugar as a victim because “sugar tastes so good it’s easy to believe it must be bad for you,” [93] a strategy similar to tobacco industry efforts to discredit public health efforts as being motived by people against “pleasure” [94, 95]. SA has also characterized the 2015–2020 U.S. Dietary Guidelines conclusions on sugars as ““opinion based” and not “science-based”” [96]. Further empirical work is required to examine whether corporate apologia theory is applicable to other sugar industry communication campaigns.

## Conclusion

Corporate apologia theory [16] can be a useful resource for the public health community and policy makers in understanding sugar industry communication strategies. Applying this theory could help identify the types of rhetorical strategies the industry will use to oppose the widespread implementation of the WHO sugars guideline. Understanding sugar industry crisis communications allows public health advocates and policy-makers to proactively plan more effective communication strategies in support of evidence-based sugar restriction policies, by preempting, rather than simply reacting to, industry messages.

## Data Availability

Data supporting the results reported in the article can be found in public library archives and online.

## Abbreviations

FDA: Food and Drug Administration
FNAC: Food and Nutrition Advisory Council
FTC: Federal Trade Commission
ISRF: International Sugar Research Foundation
NF: Nutrition Foundation
PRSA: Public Relations Society of America
SA: Sugar Association
SITDOM: Sugar in the Diet of Man
SRF: Sugar Research Foundation
WHO: World Health Organization
